# Fibrinogen: connecting the blood circulatory system with CNS scar formation

**DOI:** 10.3389/fncel.2024.1402479

**Published:** 2024-06-19

**Authors:** Pasquale Conforti, Jose C. Martínez Santamaría, Christian Schachtrup

**Affiliations:** ^1^Faculty of Medicine, Institute of Anatomy and Cell Biology, University of Freiburg, Freiburg, Germany; ^2^Faculty of Biology, University of Freiburg, Freiburg, Germany; ^3^Center for Basics in NeuroModulation (NeuroModulBasics), Faculty of Medicine, University of Freiburg, Freiburg, Germany

**Keywords:** CNS disease, blood–brain barrier opening, fibrinogen, astrocyte activation, lesion border, fibrosis, perivascular fibroblast

## Abstract

Wound healing of the central nervous system (CNS) is characterized by the classical phases of ‘hemostasis’, ‘inflammation’, ‘proliferation’, and ‘remodeling’. Uncontrolled wound healing results in pathological scar formation hindering tissue remodeling and functional recovery in the CNS. Initial blood protein extravasation and activation of the coagulation cascade secure hemostasis in CNS diseases featuring openings in the blood–brain barrier. However, the relevance of blood-derived coagulation factors was overlooked for some time in CNS wound healing and scarring. Recent advancements in animal models and human tissue analysis implicate the blood-derived coagulation factor fibrinogen as a molecular link between vascular permeability and scar formation. In this perspective, we summarize the current understanding of how fibrinogen orchestrates scar formation and highlight fibrinogen-induced signaling pathways in diverse neural and non-neural cells that may contribute to scarring in CNS disease. We particularly highlight a role of fibrinogen in the formation of the lesion border between the healthy neural tissue and the fibrotic scar. Finally, we suggest novel therapeutic strategies via manipulating the fibrinogen–scar-forming cell interaction to improve functional outcomes.

## Introduction

Tissue or organ fibrosis is the pathological scarring that occurs when the wound healing pathway is dysregulated (i.e., the appropriate healing response does not stop and becomes chronic). Pathological fibrosis is a common cause of organ failure and a leading cause of death in developed countries (Henderson et al., 2020).

Scar formation in the central nervous system (CNS) is often associated with chronic non-resolving pathology that hinders CNS repair (Silver and Miller, 2004; Cregg et al., 2014; Sofroniew, 2018; Bradbury and Burnside, 2019; Dorrier et al., 2022). The fibrotic scar is conserved across CNS injury and disease models in rodents [e.g., spinal cord injury, optic nerve injury, multiple sclerosis, and stroke (Dias et al., 2021)], suggesting that fibrosis is a common pathology in many CNS disorders. Regardless of the primary insult, fibrotic scar formation in the CNS is driven by stromal cells depositing an excess of the extracellular matrix (ECM) proteins [e.g., collagen I (Col I)] (Dias and Goritz, 2018; Dias et al., 2021; Dorrier et al., 2021, 2022; Fehlberg and Lee, 2022; Holl and Goritz, 2023). The fibrotic scar replaces lost parenchymal cells and is coordinated with the generation of the lesion border reactive astrocytes (Wanner et al., 2013; Anderson et al., 2014). However, why fibrotic scar formation is often not resolved over time and replaced by CNS parenchymal tissue is not understood. A deeper understanding of the underlying mechanisms of CNS scar formation and the cross-communication of the scar-forming cells with reactive astrocytes at the lesion border might lead to new therapeutic strategies to resolve the chronic non-resolving scar and to improve CNS repair.

Recently, our lab advanced understanding of how fibrinogen coordinates the formation of the lesion border by reactive astrocytes separating the fibrotic scar lesion center from the intact brain parenchyma (Conforti et al., 2022). Here, we will summarize our most recent findings on how the blood-derived coagulation factor fibrinogen orchestrates the formation of the astrocyte-fibroblast interface, potentially resulting in formation of a new glia limitans. This process may underlie the failure of repopulating the fibrotic scar with parenchymal cells and resolving the fibrotic lesion in the CNS over time.

### A short overview on scar formation in the CNS

The CNS lesion comprises two components. The area of focal tissue damage forms a non-neural fibrotic scar (lesion core), composed of stromal-derived fibroblasts and inflammatory cells. The surrounding penumbra consists of microglial cells and reactive astrocytes (Cregg et al., 2014). Reactive astrocytes are vital for the acute containment of inflammation and prevention of the expansion of inflammatory processes (Bush et al., 1999; Faulkner et al., 2004; Sofroniew, 2005), and these cells ultimately contribute to the chronic failure of axon regeneration (Cregg et al., 2014). Reactive astrogliosis is characterized by gradual changes in astrocytic morphology, molecular expression profile and cell proliferation with respect to distance from the lesion site. In particular, lesion border–forming astrocytes are derived from local, proliferating astrocytes (Wanner et al., 2013) and are uniquely positioned precisely at the interface to the non-neural tissue lesion core and change their physiologically defined individual domains in CNS disease (Wanner et al., 2013; Anderson et al., 2014). Neural stem/precursor cell–derived astrogenesis from the subventricular zone (SVZ) stem cell niche contributes to the reactive astrocytes in the penumbra; however, their exact morphology, localization and function at the lesion site are still not understood (Pous et al., 2020).

The central non-neural “fibrotic” lesion core is composed of stromal cells, which are restricted to vascular and meningeal niches, and CNS-associated macrophages, including perivascular macrophages and peripherally derived macrophages, which together produce an excess of ECM components, such as collagens. Different cell types are proposed to contribute to fibrotic scar formation in the CNS (e.g., type-A pericytes, and fibroblasts) (Göritz et al., 2011; Fernández-Klett et al., 2013; Soderblom et al., 2013; Kelly et al., 2016; Dias and Goritz, 2018; Yahn et al., 2019; Dias et al., 2021; Dorrier et al., 2021, 2022; Fehlberg and Lee, 2022).

### Fibrinogen instructing scar formation

The relevance of the blood circulatory system and the coagulation cascade was not considered for a long time in neuroscience, at least with regard to their roles in initiating scar formation. The wound-healing process in the CNS, such as after a stroke, typically begins with the immediate activation of the coagulation cascade to achieve hemostasis. The soluble blood-derived coagulation factor fibrinogen extravasates into the CNS parenchyma upon blood–brain barrier (BBB) disruption, is cleaved by thrombin and, upon conversion to insoluble fibrin, serves as a key matrix of blood clots to enable hemostasis (Adams et al., 2004). Beyond its role in fibrin clot formation, fibrinogen acts as a perivascular matrix to directly interact with all cellular components of the neurovascular unit to influence inflammatory, neurodegenerative and repair processes in the injured CNS (Adams et al., 2007; Schachtrup et al., 2007, 2010, 2011; Petersen et al., 2017; Pous et al., 2020).

### Fibrinogen triggers lesion border–forming astrocyte properties

In the CNS, fibrinogen acts as a multi-faceted signaling molecule by interacting with integrin and non-integrin receptors and by functioning as a carrier of growth factors (Adams et al., 2004; Martino et al., 2013; Petersen et al., 2018). We found that fibrinogen triggers astrocyte reactivity by promoting the availability of active TGF-β (Schachtrup et al., 2010) and provokes astrocyte differentiation from neural stem/precursor cells via BMP receptor I (BMP RI) signaling (Pous et al., 2020).

Recently, we reported that fibrinogen deposition guides the reactivity and morphology of astrocytes forming the lesion border (Conforti et al., 2022). Pharmacologic depletion of fibrinogen reduced the deposition of major inhibitory ECM components, such as chondroitin-sulfate proteoglycans, and resulted in a reduced astrocyte activation status. The astrocytes of fibrinogen-depleted mice revealed morphological changes (e.g., absence of flat, elongated scar border-forming astrocytes) and could penetrate the fibrotic lesion core. So far, mechanisms of how reactive astrocytes at the lesion border segregate spatially from the fibrotic lesion core are only sparsely known, but some recent results point toward the environment-dependent plasticity of reactive astrogliosis. Reactive astrocytes segregate from the lesion core via cell contact–mediated bidirectional signaling between astrocytes and fibroblasts (Ephrins and Eph receptors) and via injury-induced Plexin-B2 or type I collagen enriched in the lesion core (Bundesen et al., 2003; Hara et al., 2017; Zhou et al., 2020). Interestingly, we have discovered that fibrinogen after ischemic stroke remains deposited at the lesion border, the interface of fibrotic cells and reactive, border-forming astrocytes, potentially preventing the parenchymal cell penetration into the fibrotic scar. Fibrinogen depletion reduced injury-induced Plexin-B2 expression in the lesion core, suggesting that fibrinogen orchestrates the environment-dependent plasticity of astrocytes and fibrotic cells at the lesion border. We propose that the initial hemostasis and massive fibrinogen deposition in the CNS parenchyma is a major driver and organizer of scar formation and that prolonged fibrinogen deposition at the lesion border establishes the segregation between parenchymal astrocytes and fibrotic cells in the CNS.

### Glia limitans and lesion border – similarities and differences

The glia limitans or the glial limiting membrane is a thin barrier of astrocyte foot processes associated with the parenchymal basal lamina surrounding the brain and spinal cord. It is the outermost layer of neural tissue, and among its responsibilities is the prevention of the over-migration of neurons and neuroglia, the supporting cells of the nervous system, into the meninges.

**Figure 1:**
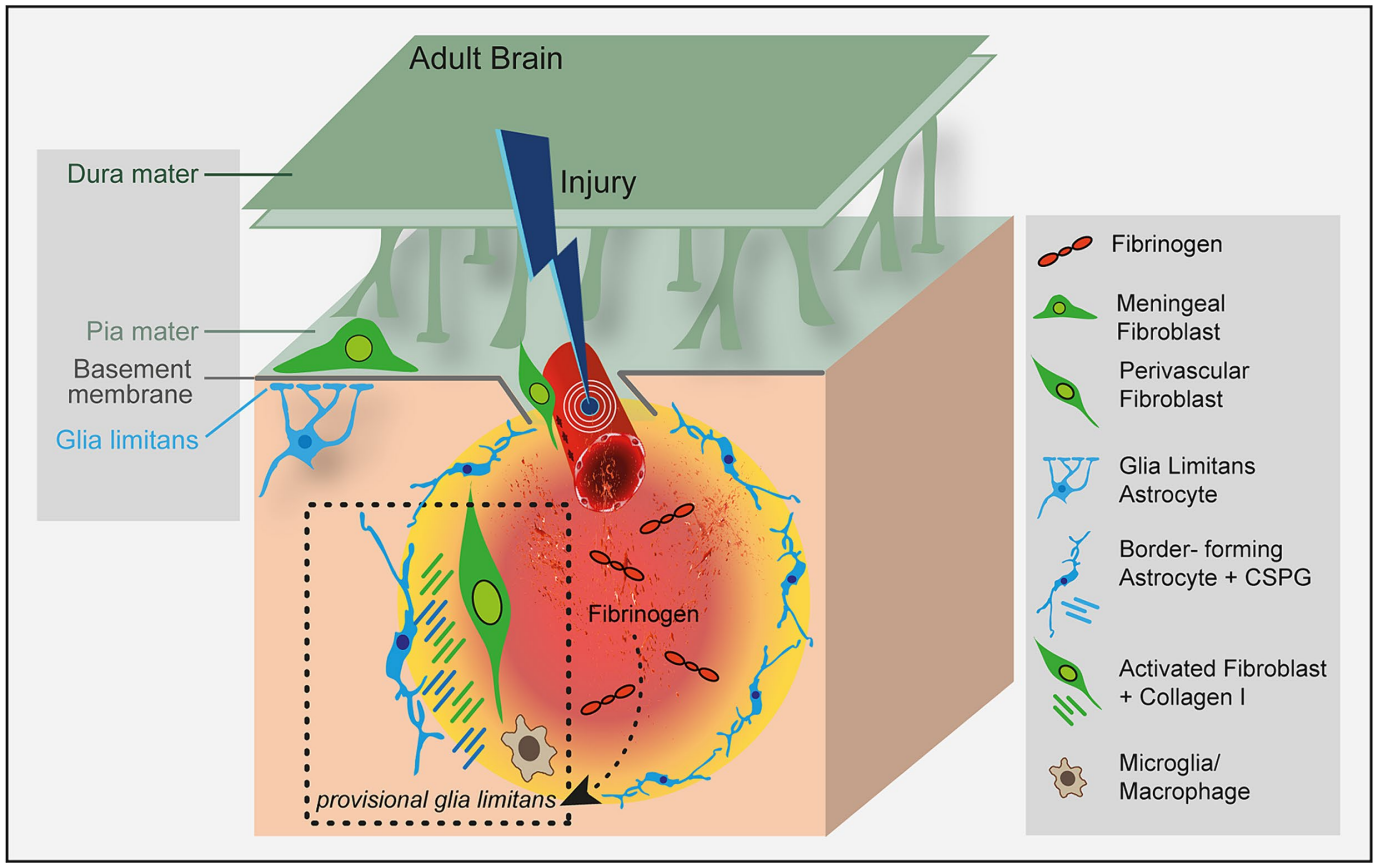
Fibrinogen regulates lesion border formation at the astrocyte-fibroblast interface. Fibrinogen triggers astrocyte reactivity by promoting the availability of active TGF-β (Schachtrup et al., 2010) and altering their morphology and functionality (Conforti et al., 2022) to form the perilesional border. The effects of fibrinogen on mesenchymal cells that contribute to fibrotic scarring have not been studied so far. Fibrinogen might connect the blood circulatory system with CNS scar formation by orchestrating the formation of a provisional glia limitans in CNS disease with BBB openings.

The development of the long astrocyte cellular processes that are integral to the glia limitans structure has been linked to the presence of meningeal cells in the pia mater (Struckhoff, 1995). Astrocytes are bushy star-shaped glial cells in the CNS that, like neurons and oligodendrocytes, arise from a radial glial cell population of ectodermal, neuroepithelial origin surrounding the ventricular zone in the early developing brain (Rowitch and Kriegstein, 2010). In proximity to the pia mater, astrocytes send out processes to form the glia limitans superficialis (Figure 1) (Struckhoff, 1995). A thin barrier of astrocyte endfeet processes is associated with the parenchymal basal lamina via the dystrophin-associated glycoprotein complex, connecting the astrocyte basal membrane to the astrocyte cytoskeleton (Jiang et al., 2002). Fluid and low-molecular-weight tracers pass freely through the glia limitans from the CSF (Iliff et al., 2012), but trafficking of immune cells, such as T cells, is limited (Howell et al., 2015).

Meningeal fibroblasts in the midbrain, hindbrain and spinal cord originate from the somatic and cephalic mesoderm, whereas those in the forebrain originate from the neural crest (Couly et al., 1992; Jiang et al., 2002; Yoshida et al., 2008). Intensive signaling events between meningeal fibroblasts and the brain regulate cortical development and the formation of the glia limitans superficialis (e.g., pia mater fibroblasts begin to secrete leukemia inhibitory factor, retinoic acid, and BMP4 to initiate astrogliogenesis at E14, forming the glia limitans at the boundaries of the meninges) (Kawamura et al., 2017; Katada et al., 2021). Additionally, astrocyte endfeet form the glia limitans perivascularis, which line the CNS side of the blood brain barrier (BBB) to further separate the CNS from the peripheral circulation; this provides an additional physical barrier to keep peripheral immune cells out of the CNS.

Interestingly, perivascular fibroblasts (PVF) located on the outside of the pericyte or vascular smooth muscle cell (vSMC) layer in the perivascular space of large-diameter arterioles and venules, but not capillaries, oppose the astrocyte endfeet (Figure 1). With the advent of single-cell RNA sequencing (scRNA-seq) technologies, it has become increasingly recognized that PVF show enriched expression for ECM structural components and its modifiers or receptors, suggesting a function in the astrocytic basement membrane formation (Yang et al., 2022). However, whether PVF and astrocytes communicate to form the glia limitans perivascularis is so far unknown. Intensive research has focused on the robust response of astrocytes and fibrotic cells in CNS disease, summarized in excellent recent review articles (Dorrier et al., 2022; Fehlberg and Lee, 2022). However, how astrocytes and fibroblasts communicate and which molecules affect their interaction to form a lesion border in CNS disease are largely unknown.

Lesions in the brain trigger formation of the fibrotic scar in parallel and in coordination with reactive astrogliosis. The resulting interface between the CNS parenchymal tissue and the fibrotic lesion has similarities in appearance and function to the glia limitans formed by perimeningeal astrocytes that interface with stromal cells of the meninges around the entire CNS (Sofroniew, 2020; Wahane and Sofroniew, 2022). A physicochemical barrier composed of chondroitin sulfate proteoglycans, collagen I and dense extracellular matrix behaves as a basement membrane located at the interface between the reactive astrocyte-formed lesion border and the fibrotic scar (Figure 1). After a brain lesion, the breach of the BBB immediately initiates the coagulation cascade and a drastic change in the ECM by deposition of the provisional fibrin matrix (Schachtrup et al., 2007). The provisional fibrin matrix triggers reactive responses of neural parenchymal cells, most notably reactive astrogliosis (Schachtrup et al., 2010; Pous et al., 2020; Conforti et al., 2022) and microgliosis (Adams et al., 2007; Ryu et al., 2018). However, the contribution of fibrinogen to the complex picture of the forming barrier, its underlying molecular mechanisms of the genesis, and its implications for brain repair are only marginally understood.

### Fibrinogen – bridging neural and mesenchymal tissue

As we strive to understand the biological complexity of scar tissue formation and tissue fibrosis in the CNS, we clearly cannot study glia, non-neural stromal cells, and the ECM in isolation. A first key change in the molecular composition of the extracellular microenvironment in CNS disease is the abundant extravasation of the plasma protein fibrinogen into the CNS parenchyma through a leaky BBB. Given its pleiotropic functions, fibrinogen may be an apical signal that orchestrates the molecular and cellular composition of the CNS after vascular damage affecting neural and mesenchymal cells to coordinate scar formation.

Human fibrinogen is made up of three pairs of polypeptide chains, namely Aα, Bβ and γ (Lord, 2011). Each chain contains Arg-Gly-Asp (RGD) sequences that serve as attachment sites for multiple integrins and receptors (Petersen et al., 2018). Besides, the αC domains can bind latent TGF-β (Schachtrup et al., 2010) and the dimeric sequence (β15-66)_2_, which is exposed after fibrinogen conversion into fibrin, binds heparin and growth factors from the PDGF/VEGF, FGF, TGF-β and neurotrophin families (Martino et al., 2013).

**Figure 2:**
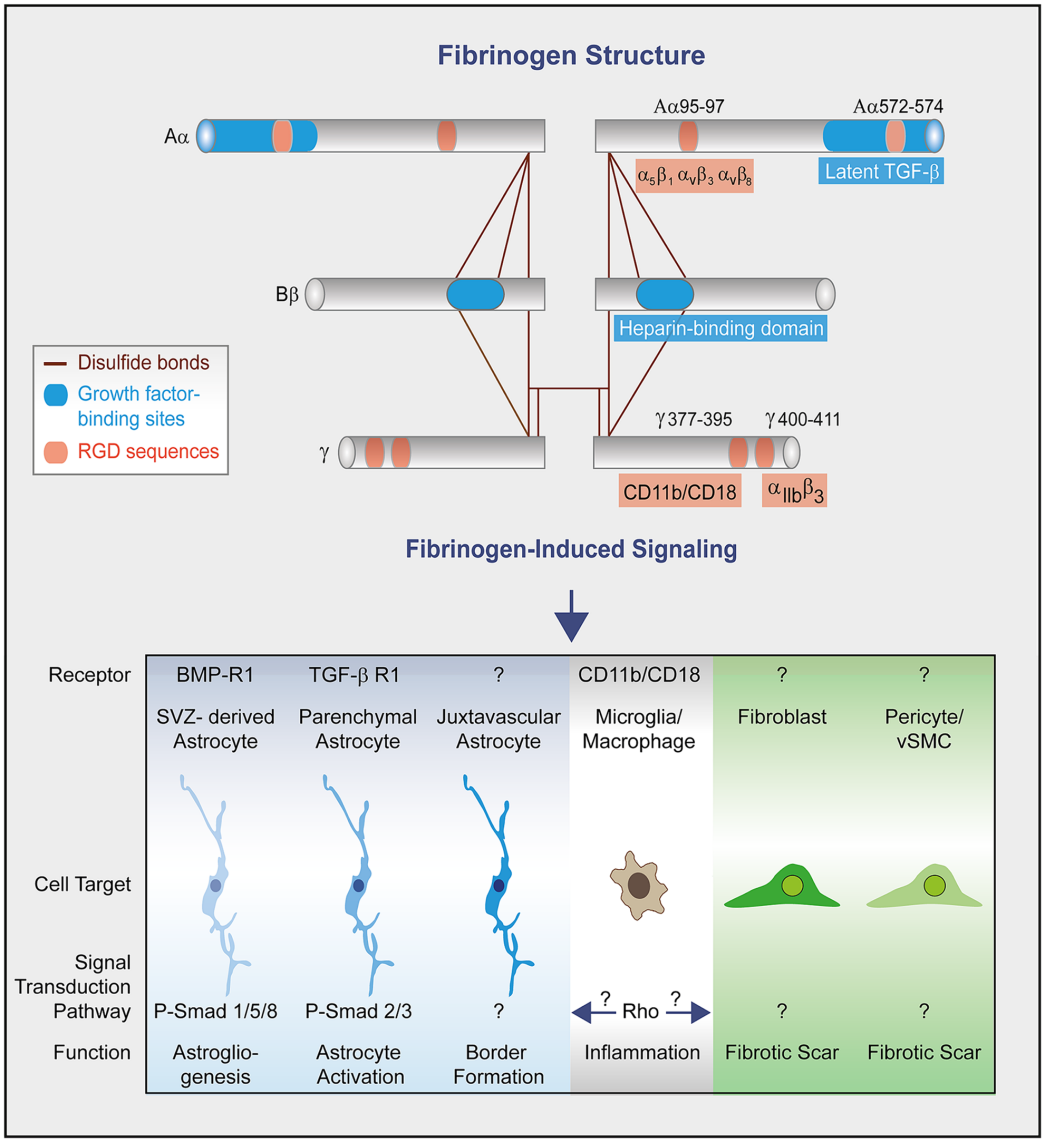
Fibrinogen structure and signaling in scar formation. Fibrinogen is a dimer composed of three distinct polypeptidic chains (Aα, Bβ, and γ) bound by disulfide bonds (Lord, 2011). Each chain contains both RGD sequences and binding sites for growth factors. Fibrinogen chain Aα binds, among other, latent transforming growth factor-β (TGFβ) (Schachtrup et al., 2010) and interacts with the integrin receptors α5β1, αvβ3 and αvβ8 through its Arg-Gly-Asp (RGD) motifs (Smith et al., 1990; Suehiro et al., 2000; Chernousov and Carey, 2003). The dimeric chain Bβ sequence (β15-66)_2_ in fibrin (heparin binding domain) binds heparin and growth factors from the PDGF/VEGF, FGF, and TGF-β families (Martino et al., 2013). Fibrinogen chain γ binds the integrin receptors CD11b/CD18 (Ugarova et al., 2003) and α_IIb_β_3_ (Weisel, 2005). Fibrinogen activates growth factor receptor pathways in SVZ-derived astrocytes and parenchymal astrocytes to regulate astrogliogenesis and astrocyte activation, respectively (Schachtrup et al., 2010; Pous et al., 2020). The fibrinogen-induced signal pathway to regulate the morphology and functionality of juxtavascular astrocytes to form the lesion border (Conforti et al., 2022) is unknown so far. The conversion of fibrinogen into fibrin exposes cryptic epitopes, such as the γ377–395 epitope, which is the binding site for the CD11b/CD18 integrin receptor and activates microglia/macrophages (Adams et al., 2007). Fibrinogen-induced microglia/macrophage activation might in turn trigger activation of scar forming cells. The effects of fibrinogen on mesenchymal cells (e.g., fibroblasts, pericytes and vascular smooth muscle cells) leading to fibrotic scar formation remain unknown.

Fibrinogen induces growth factor receptor pathway activation in parenchymal astrocytes and SVZ-derived newborn astrocytes to regulate scar formation and astrogliogenesis, respectively (Figure 2; Schachtrup et al., 2010; Conforti et al., 2022). Our studies revealed that fibrinogen triggers parenchymal astrocyte activation in a TGF-β type I receptor-dependent manner and that fibrinogen induces SVZ-derived Thbs4 + NSPC differentiation into newborn reactive astrocytes as well as the formation of astrocyte-like cells from NG2 + oligodendrocyte progenitor cells in a BMP RI–dependent manner. However, depletion of fibrinogen did not affect the proliferation and formation of new (Ki67+/EdU+) local, juxtavascular astrocytes at the lesion site, suggesting that in the lesion area fibrinogen deposition does not affect the overall astrocyte cell number, but its activation status. The fibrinogen-induced changes in morphology and functionality of the different astrocyte subtypes that contribute to repair processes still need to be determined. Furthermore, how fibrinogen exerts biological effects and signaling on mesenchymal cells, including meningeal and perivascular fibroblasts, pericytes and vSMCs to contribute to CNS fibrotic scarring, has not yet been studied (Figure 2).

Microglia, monocyte-derived macrophages, and immune cell infiltrates are also intimately involved in the scarring process (Bellver-Landete et al., 2019; Li et al., 2020; Zhou et al., 2020; Dorrier et al., 2021). Conversion of fibrinogen into fibrin exposes cryptic epitopes, such as the γ377–395 epitope, which is the binding site for the CD11b/CD18 integrin receptor, inducing microglia and macrophage activation upon receptor binding (Figure 2; Adams et al., 2007). Therefore, fibrin-induced microglia and macrophage activation might affect cell activation and function of other scar forming cells, such as astrocytes, pericytes, and vSMCs, as well as meningeal and perivascular fibroblasts.

Thus, blood-derived fibrinogen with its unique function to form a provisional matrix might instruct neural and mesenchymal tissue to orchestrate scar formation and to connect tissues of different origin at the lesion border potentially forming a new glia limitans.

## Conclusion

The molecular triggers for development of the lesion border by reactive astrocytes and the fibrotic scar are poorly described in CNS diseases. However, fibrinogen, which is immediately and massively deposited at the lesion site and which, due its unique molecular structure to interact with the different neural and non-neural cell types, may orchestrate wound healing in the CNS. Cutting-edge techniques to determine the fibrinogen-induced transcriptome and secretome of scar-forming cells over time will provide fundamental information on fibrinogen-driven cell–cell communication in the lesion area and will improve understand of how wound healing in the CNS is organized. Furthermore, genetic or pharmacologic approaches to deplete fibrinogen might be potent tools for understanding how fibrinogen affects the different wound-healing phases hemostasis, inflammation, proliferation, and remodeling in the CNS. The manipulation of fibrinogen-induced molecular cascades on specific cell types driving pathological scarring might create an environment that is more beneficial for promoting repair processes. This was recently shown for a newly generated monoclonal antibody that selectively inhibits fibrin-induced inflammation and oxidative stress without interfering with clotting (Ryu et al., 2018). We hope to foster research into the nature and development of neural-mesenchymal interfaces, with the aim of designing strategies that resolve fibrotic scarring and improve the functional outcome in CNS disease.

## Data availability statement

The original contributions presented in the study are included in the article/supplementary material, further inquiries can be directed to the corresponding authors.

## Ethics statement

The animal study was approved by Federal Ministry for Nature, Environment and Consumers Protection of the state of Baden-Württemberg. The study was conducted in accordance with the local legislation and institutional requirements.

## Author contributions

PC: Conceptualization, Writing – original draft. JM: Conceptualization, Writing – original draft. CS: Conceptualization, Writing – original draft.
